# The Clinical Anatomy of SARS-CoV-2 Variants of Concern in Central Greece During October 2020–July 2022

**DOI:** 10.3390/microorganisms12122573

**Published:** 2024-12-13

**Authors:** Ioanna Voulgaridi, Zacharoula Bogogiannidou, Katerina Dadouli, Achilleas P. Galanopoulos, Maria A. Kyritsi, Alexandros Vontas, Alexia Matziri, Konstantina Kola, Evangelia Vachtsioli, Lemonia Anagnostopoulos, Anastasia Tsispara, Katerina G. Oikonomou, Dimitris Babalis, Efthymia Petinaki, Maria Tseroni, Fani Kalala, Matthaios Speletas, Varvara A. Mouchtouri, Christos Hadjichristodoulou

**Affiliations:** 1 Laboratory of Hygiene and Epidemiology, Faculty of Medicine, University of Thessaly, 413 34 Larissa, Greece; ioavoulg@uth.gr (I.V.); zbogogiannidou@uth.gr (Z.B.); adadouli@uth.gr (K.D.); acgalanopoulos@uth.gr (A.P.G.); mkiritsi@uth.gr (M.A.K.); avontas@uth.gr (A.V.); alexmatz@uth.gr (A.M.); kokola@uth.gr (K.K.); evachtsioli@uth.gr (E.V.); lanagnost@uth.gr (L.A.); mouchtourib@uth.gr (V.A.M.); 2 Department of Immunology and Histocompatibility, Faculty of Medicine, University of Thessaly, 413 34 Larissa, Greece; fkalala@uth.gr (F.K.); maspel@uth.gr (M.S.); 3 Emergency Department, General Hospital of Larissa, 413 34 Larissa, Greece; tsispara@gmail.com (A.T.); dbabales@yahoo.com (D.B.); 4 Intensive Care Unit, General Hospital of Larissa, 413 34 Larissa, Greece; oikonomoukaterina85@gmail.com; 5 Department of Microbiology, University Hospital of Larissa, University of Thessaly, 413 34 Larissa, Greece; petinaki@uth.gr; 6 Department of Nursing, School of Health Sciences, National and Kapodistrian University of Athens, 157 72 Athens, Greece; mtseroni@nurs.uoa.gr

**Keywords:** SARS-CoV-2, variants of concern (VOCs), outcomes of COVID-19, differences in symptomatology, differences in severity, vaccination

## Abstract

The emergence of SARS-CoV-2 variants of concern (VOCs) during the COVID-19 pandemic necessitates investigation into their clinical differentiation and outcomes. This study aimed to examine these differences among VOCs, considering multiple related factors. An observational cohort study was conducted on patients diagnosed with SARS-CoV-2 infection via nasopharyngeal/oropharyngeal swab who visited the emergency department of a public Greek hospital between October 2020 and July 2022 during different VOC circulation in the region. Data on clinical manifestations, outcomes, and medical history (comorbidities, prior SARS-CoV-2 infection, vaccination status against COVID-19) were collected through a questionnaire and medical records for those hospitalized. A total of 913 patients were included in this study (813 adults ≥18 years old, 100 children <18 years old). Significant differences were observed across VOCs for both adults and children. A lower proportion of children developed symptoms during the non-Omicron variants, 73.5%, compared to Omicron variants, 86.4%. Fever, dyspnea, and taste and smell disorders were observed more frequently among non-Omicron adult cases, in contrast to upper respiratory symptoms, which were more common symptoms among Omicron infections. The non-Omicron variants were associated with higher rates of hospitalization at 30.6%, pneumonia at 23.0%, and death at 6.1% compared to Omicron variants at 8.0%, 5.0%, and 1.8%, respectively. Vaccination against COVID-19 was shown to be a protective factor for severe outcomes. Our findings suggest distinct clinical presentations and outcomes associated with different VOCs. Despite the fact that current VOCs circulating are less severe, the COVID-19 vaccine continues to play a protective role for severe cases.

## 1. Introduction

On 31 December 2019, the first cluster of coronavirus disease 2019 (COVID-19) cases was declared in Wuhan, Hubei Province, China, as cases of pneumonia of unknown etiology. Twelve days later, the virus’s genetic sequence was announced and classified in the genus Beta coronavirus.

Similar to other RNA viruses, through mutation of its genetic elements, severe acute respiratory syndrome coronavirus 2 (SARS-CoV-2) has the ability to evolve, resulting in the diversification of characteristics such as environmental survival, contagiousness, and pathogenicity. SARS-CoV-2 mutations led to the emergence of multiple variants, with different characteristics compared to their ancestral strain, wild type (WT) (Wuhan Hu 1) [1]. Just one alteration in an amino acid could significantly impact the virus’s capacity to evade the immune system, nullifying an existing effective vaccine [1]. The World Health Organization (WHO) declared that certain variants were of higher concern due to increased risk and greater impact on public health; this prompted the characterization of specific variants of interest (VOIs) [2]. According to the WHO, a SARS-CoV-2 variant is characterized as a VOI when the first genetic changes are predicted or known to affect virus characteristics, such as transmissibility, disease severity, immune escape, diagnostic or therapeutic escape, and when it causes significant community transmission or multiple COVID-19 clusters in multiple countries, with increasing relative prevalence alongside an increasing number of cases over time or other apparent epidemiological impacts that suggest an emerging risk to global public health [2]. A SARS-CoV-2 variant is characterized as a variant of concern (VOC) when it meets the definition of a VOI and through comparative assessment has a demonstrated association with one or more of the following changes of global public health significance: (1) an increase in transmissibility or detrimental change in COVID-19 epidemiology; (2) an increase in virulence or change in clinical disease presentation; and (3) a decrease in the effectiveness of public health and social measures (PHSMs) or available diagnostics, vaccines, or therapeutics [2].

As of July 2022, nine VOCs were identified from the start of the pandemic. Alpha (B.1.1.7) was the first VOC described in the United Kingdom (UK) in late December 2020; this was followed by Beta (B.1.351), first reported in South Africa in December 2020; Gamma (P.1.), initially documented in November 2020 in Brazil; Delta (B.1.617.2), first reported in India in December 2020; Omicron BA.1 (B.1.1.529.1), initially reported in South Africa in November 2021; and Omicron BA.2 (B.1.1.529.2), Omicron BA.3 (B.1.1.529.3), Omicron BA.4 (B.1.1.529.4), and Omicron BA.5 (B.1.1.529.5). VOCs are associated with enhanced transmissibility or virulence, a reduction in neutralization by antibodies obtained through natural infection or vaccination, an ability to escape the immune system, or a decrease in effective of therapeutics or vaccination [1,3].

The clinical impact of VOCs varies, with multiple factors seeming to play an important role in COVID-19 severity. Our study aims to identify differences in the clinical image and outcomes of COVID-19 among different SARS-CoV-2 VOCs by considering the demographic variables of participants, their underlying diseases, and medical history of previous SARS-CoV-2 infection or vaccination against COVID-19.

## 2. Materials and Methods

### 2.1. Study Design and Participants

An observational cohort study was conducted in a region of central Greece from October 2020 to July 2022. Our participants were derived from individuals who tested positive for SARS-CoV-2 infection from nasopharyngeal/oropharyngeal samples. These individuals had presented to a public hospital emergency department due to symptoms compatible with COVID-19 or close contact with a confirmed COVID-19 case. Participants were randomly selected among laboratory-confirmed cases and were contacted to obtain information regarding symptoms and outcomes. During the study design phase, we aimed to have an equal number of participants in each variant group. Matching among participants was conducted to adjust for age and sex.

This study utilized a closed-ended telephone or paper-based questionnaire to gather information about participants’ demographic data, comorbidities, clinical symptoms due to COVID-19 (fever, cough, sore throat, nasal congestion or runny nose, headache, fatigue, taste or smell disorders, gastrointestinal disorders, dyspnea, etc.) and treatment, SARS-CoV-2 infection outcome (diagnosis of pneumonia, hospitalization, admission to high-dependency care units (HDUs), admission to intensive care units (ICUs), intubation, death), and medical history of previous SARS-CoV-2 infection. Telephone interviews were conducted fifteen days following the diagnosis of SARS-CoV-2 infection. In case of persistent symptomology, interviews were repeated one month following the first interview. Information regarding hospitalized patients or participants with a history of hospitalization due to COVID-19 was obtained from their medical records. Following the implementation of the national vaccination strategy, information was also collected regarding vaccine administration, vaccination date, vaccine type, and number of doses.

### 2.2. Laboratory Analysis

Respiratory samples (nasopharyngeal or oropharyngeal swabs) were collected in transfer tubes containing viral transport medium. Samples were analyzed at the Laboratory of Hygiene and Epidemiology, Faculty of Medicine, University of Thessaly, which functioned as a Public Health Laboratory to address the heightened demands resulting from the COVID-19 pandemic. SARS-CoV-2 RNA was isolated from samples with the KingFisher Flex System (ThermoFisher Scientific, Waltham, MA, USA) using the MagMAX™ Viral/Pathogen Nucleic Acid Isolation Kit (Applied Biosystems™, Waltham, MA, USA) according to the manufacturer’s instructions. Detection of the virus’s genetic material was performed using reverse transcription quantitative real-time polymerase chain reaction (RT-qPCR), with primers targeting SARS-CoV-2-specific genes: ORF1ab (Open Reading Frames), N protein (Nucleocapsid protein), and S protein (Spike protein) with the TaqPath™ COVID-19 CE-IVD RT-PCR Kit (Applied Biosystems™, Waltham, MA, USA) on a validated QuantStudio™ 5 Real-Time PCR System (ThermoFisher Scientific, Waltham, MA, USA). The threshold for positivity ≤37 cycle threshold (Ct) for SARS-CoV-2 infection was established.

Participants’ classification by VOC was first screened using as a marker the detection or absence of the S gene via RT-qPCR, known as “S gene dropout” or “spike gene target failure” [4,5,6]. During the study period, a proportion of the total number of each variant was confirmed with Next-Generation Sequencing (NGS). The NGS analysis was performed with the Illumina COVIDSeq™ Assay (Illumina Inc. San Diego, CA, USA). (Research Use Only (RUO)) (96 samples), which incorporates the ARTIC v4.0 multiplex PCR protocol on a NextSeq2000 sequencing system (Illumina Inc. San Diego, CA, USA). To identify SARS-CoV-2 variants, Illumina’s DRAGEN COVID Lineage App in BaseSpace™ (pipeline and lineage tool) (RUO) v1.3.0 was used.

RT-qPCR tests and the majority of NGS sample analyses were conducted at the Laboratory of Hygiene and Epidemiology, Faculty of Medicine, University of Thessaly, located in Larissa, Greece. The remaining NGS sample analyses were performed at the central laboratory of the National Public Health Organization (NPHO) in Athens, Greece.

### 2.3. Classification of COVID-19 Severity and Vaccination Status

We categorized the severity of COVID-19 cases into four classes: (a) severity 0: no referred symptoms; (b) severity 1: mild symptoms; (c) severity 2: severe disease (hospitalization, including HDU admission and excluding ICU admission); and (d) severity 3: critical care patients (ICU admission, intubation) or death caused by SARS-CoV-2 infection.

Participants were considered fully vaccinated with at least the minimum doses required for complete immunization, according to manufacturers’ directions and guidelines from the NPHO for each vaccine type.

### 2.4. Statistical Analysis

A total of 180 samples were estimated for each VOC, considering the anticipated proportion of individuals who may decline the interview or not respond. Categorical variables were described using frequency and relative frequency, while continuous variables were described using medians and interquartile range (IQR). Associations between categorical variables were tested using the chi-squared test, while the Mann–Whitney or Kruskal–Wallis tests were used to examine associations between categorical and continuous variables. Data were checked for deviation from normal distribution using the Shapiro–Wilk normality test. The “variants” were used as 6-value variables as well as binary variables, as defined by the number of participants related to the under-study outcome. To estimate the effect of age, sex, comorbidities, predominant variant, and vaccination on disease outcomes, we used odds ratios (ORs) with a 95% confidence interval (CI), estimated with binary logistic regression models. Furthermore, to compare the relative incidence of each symptom between one variant and the remaining, we also used ORs with a 95% CI estimated with binary logistic regression models. These models were adjusted for age, sex, and vaccination status. For all analyses, a 5% significance level was set. Analysis was carried out with R language (version 4.3.1) [7]. “Table1”, “sjPlot”, “tidyverse”, “ggplot2” (version 3.5.0) packages were employed to conduct the analysis [8]. 

### 2.5. Ethical Statement

The research protocol was approved by the Research Ethics Committee of the Faculty of Medicine, University of Thessaly, Greece (84/09.12.2022), and was also approved by the Scientific Council of the public hospital from which the sample was derived (19535/28.06.2023).

## 3. Results

### 3.1. Characteristics and Clinical Image of Participants with Laboratory-Confirmed SARS-CoV-2 Infection

Between October 2020 and July 2022, from a total of 1075 randomly selected individuals, 913 responded positively and participated in this study (response rate 85%).

#### 3.1.1. Demographic Characteristics of Children (<18 Years Old) with Laboratory-Confirmed SARS-CoV-2 Infection

Among 100 minor participants, 60 (60%) were male; their ages ranged from 2.5 months to 17 years old (median: 11 years, IQR: 8–13 years). Of the total number of children, thirty-four were infected by a non-Omicron variant: eleven by WTS, eight by the Alpha variant, and fifteen by the Delta variant. Meanwhile, 66 were infected by the Omicron variant, with 30 cases attributed to Omicron BA.1, 16 to Omicron BA.2, and 20 to Omicron BA.4/ΒA.5. Table S1 details the medical history of minor participants, including their vaccination status against COVID-19 and/or previous SARS-CoV-2 infection.

#### 3.1.2. Characteristics of Symptoms Due to COVID-19 in Children (<18 Years Old)

As presented in Table 1, 82 children developed symptomatology (82%). The proportions of symptomatic infected children showed a statistically significant difference among VOCs (*p* = 0.039). Children infected by Omicron variants developed more frequent symptoms compared to non-Omicron variants (Omicron: 86.4%, non-Omicron: 73.5%), with Omicron BA.4/BA.5 and Omicron BA.2 presenting the two highest proportions of symptomatic cases (ΒA.4/BA.5: 95%, BA.2: 93.8%). Fever, nasal congestion/discharge, and cough were the most common symptoms, with fever developing in 62 of 100 children (62%), nasal congestion/discharge in 37 children (37%), and cough in 36 children (36%). The presence of fever was more common for the Omicron BA.2 and Delta variants (14/16 (87.5%) and 12/15 (80%)) compared to the remaining variants (*p* = 0.046). The longest duration of fever recorded was three days, referring to cases of infection by the Delta variant. As presented in Table 1, symptoms caused by upper respiratory tract infection such as cough, nasal congestion/discharge, and sore throat were primarily observed in cases of Omicron variant infection; 26 of 66 Omicron cases presented cough (39.4%), 31 developed nasal congestion/discharge (47%), and 19 experienced sore throat (28.8%). Smell and taste disorders were recorded more frequently among infections by the Delta variant, observed in five of fifteen cases (33.3%). Although clinical manifestations from the gastrointestinal (GI) tract were more commonly reported among Omicron cases (Omicron: 10.6% versus non-Omicron: 2.9%), a statistically significant difference was not identified among the VOCs (*p* = 0.662). An overview of the clinical picture is detailed in Table 1. Among the pediatric population studied, no cases with severity 3 and few cases from each variant were characterized as severity 2 (Table 1).

#### 3.1.3. Demographic Characteristics of Adults (≥18 Years Old) with Laboratory-Confirmed SARS-CoV-2 Infection

In total, 813 adults (≥18 years old) participated in this study, and their ages ranged from 18 to 100 years old (median: 47 years old, IQR: 35–60 years old), with 464 of them being female (57.1%). Descriptive data about sex, age, comorbidities, medical history of previous SARS-CoV-2 infection, and vaccination per VOC are cited in Table S2.

#### 3.1.4. Characteristics of Symptoms Due to COVID-19 in Adults (≥18 Years Old)

Among all adult participants, 762 reported experiencing at least one symptom (93.7%). No statistically significant difference was found in the proportions of COVID-19 cases developed clinical manifestations among VOCs (*p* = 0.813). The duration of symptoms was observed to last longer in cases of non-Omicron variants compared to Omicron variants (*p* < 0.001) (Table 2). Regarding symptom duration, fourteen Delta cases (9.9%) and fifteen Alpha cases (9.6%) reported symptoms lasting longer than 20 days, while eight WTS cases (4.5%) and two Omicron cases (0.8%) reported durations exceeding 20 days.

Table 2 presents the total number of symptoms and their frequency for each variant. Fever, dyspnea, and taste and smell disorders were observed more frequently among non-Omicron cases, in contrast to sore throat and nasal congestion/discharge, which were more common symptoms among Omicron infections. From the total number of cases infected by non-Omicron variants (N = 474), 381 experienced fever (80.4%), 111 experienced dyspnea (23.4%), 164 experienced taste disorders (34.6%), and 146 experienced smell disorders (30.8%). In comparison, among Omicron-infected cases (N = 339) 226 presented fever (66.7%), 27 experienced breathing difficulties (8.0%), 45 experienced taste disorders (13.3%), and 37 experienced smell disorders (10.9%). Regarding the most commonly developed symptoms among Omicron variant infections, 139 cases experienced sore throat (41.0%) and 168 cases reported nasal congestion/discharge (49.6%). In contrast, among non-Omicron variant infections, only 98 cases experienced sore throat (20.7%) and 101 cases reported nasal congestion/discharge (21.3%).

**Figure 1:**
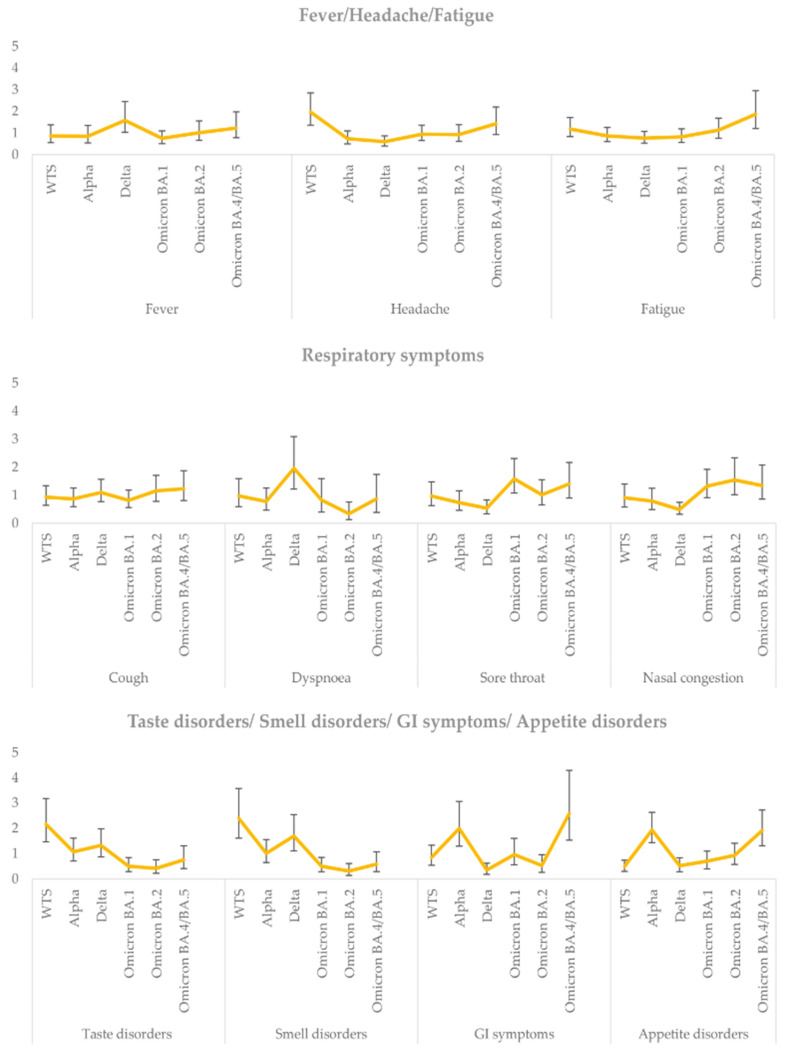
Comparison of ORs ^1^ for positivity in each variant versus the rest variants based on the presence or absence of any symptoms surveyed in SARS-CoV-2 ^2^-positive participants (Note: ORs are derived from logistic regression models for positivity in each variant versus the rest variants (1/0) as the outcome variable, adjusted for age, sex, and vaccination status. Error bars show 95% confidence intervals). ^1^ OR: odds ratio; ^2^ SARS-CoV-2: severe acute respiratory syndrome coronavirus 2.

Figure 1 presents the OR for developing the symptom under study in each variant versus the remaining VOCs. Fever and dyspnea were more common among the Delta variant (OR = 1.58 and OR = 1.96, respectively). Symptoms from the upper respiratory system (sore throat and nasal congestion) appeared more frequently in the Omicron VOCs. Taste and smell disorders were primarily observed among non-Omicron variants, particularly WTS and Delta (WTS: taste disorders OR = 2.16 and smell disorders OR = 2.39; Delta: taste disorders OR = 1.32 and smell disorders OR = 1.69). As presented in Figure 1, GI symptoms and appetite disorders were more likely to have been reported in infections by the Alpha and Omicron BA.4/BA.5 variants (Alpha: GI OR = 1.99 and appetite disorders OR = 1.93, Omicron BA.4/BA.5: GI OR = 2.58 and appetite disorders OR = 1.91).

### 3.2. Total Findings Regarding Clinical Manifestations in the Entire Study Population

**Figure 2:**
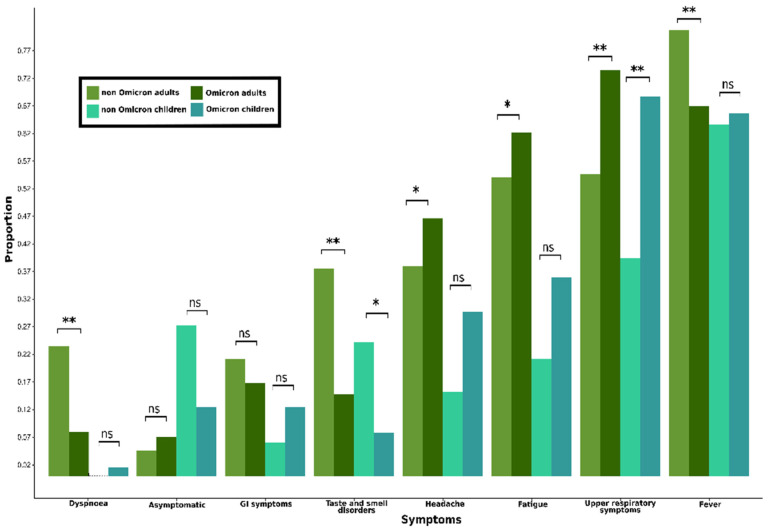
Comparison of clinical manifestations in children and adults among Omicron and non-Omicron cases (Note: ns: non-significant; *: 0.001< *p* < 0.05; **: *p* ≤ 0.001).

Figure 2 illustrates the comparison of symptoms experienced by adults and children. Fever and dyspnea were more common among adults infected by non-Omicron than by Omicron variants (fever: OR = 2.05, *p* < 0.001, dyspnea: OR = 3.53, *p* < 0.001), while headache and fatigue were more frequent among Omicron adult cases (headache: OR = 1.43, *p* = 0.012, fatigue: OR = 1.41, *p* = 0.011). In both age groups, upper respiratory symptoms were more common in Omicron cases (adults OR = 0.44 with *p* < 0.001, and children OR = 0.31 with *p* = 0.001). Olfactory and gustatory disorders were more commonly reported in non-Omicron variants (adults: OR = 3.44, *p*< 0.001, children: OR = 5.05, *p* = 0.024). In Figure S1, the percentage proportion and the course of the most common symptoms reported by the entire study population, including adults and children, are presented.

### 3.3. Severity of COVID-19 in Adults (≥18 Years Old)

**Figure 3:**
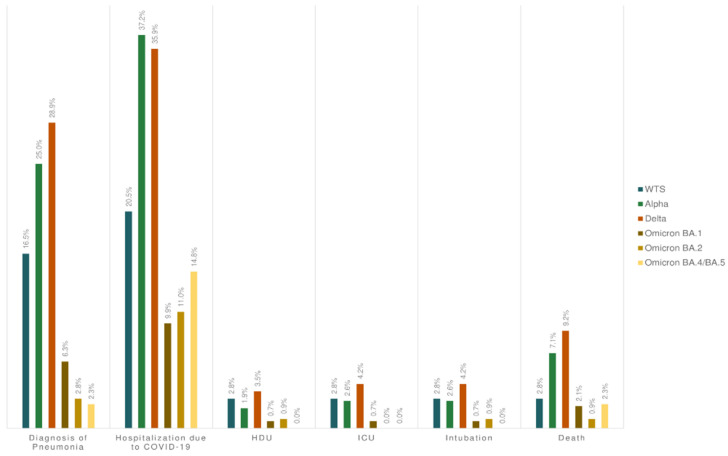
The proportion (%) of severe outcomes of COVID-19 per SARS-CoV-2 VOC during October 2020–July 2022.

Table 3 presents the outcomes of COVID-19 among adults categorized as severity 2 or 3—there is no respective section for children due to the low number of reported cases classified as severity level 2 and the complete absence of cases classified as severity level 3. The Alpha and Delta variants were responsible for a higher number of hospitalizations, specifically 58 of 156 (37.2%) and 51 of 142 (35.9%), respectively (*p* < 0.001). These two variants caused pneumonia and death more frequently than the remaining VOCs (pneumonia: Delta: 41/142 (28.9%) and Alpha: 39/156 (25.0%), *p* < 0.001; death: Delta: 13/142 (9.2%) and Alpha: 11/156 (7.1%), *p* = 0.004). HDU admission, ICU admission, and intubation were recorded more frequently in Delta and WTS, without a statistically significant difference. The frequency of COVID-19 outcomes categorized as severity 2 or 3 is provided in Figure 3.

The association between specific risk factors and the risk of outcomes in adults with severity 2 or 3 was examined through multivariate analysis (Table S3). For each outcome of severity 3, multivariate analysis was restricted to non-Omicron cases due to few Omicron patients. Age was estimated as a risk factor for every critical outcome. Vaccination was identified as a stable protective agent for hospitalization and pneumonia in both non-Omicron and Omicron variants (*p* < 0.001). Immunization via vaccine appeared to reduce the possibility of ICU admission and intubation in non-Omicron cases (ICU admission: OR: 0.16, *p* = 0.098; intubation: OR: 0.15, *p* = 0.085). The risk of death among vaccinated cases infected by a non-Omicron variant was approximately five times lower compared to unvaccinated cases infected by the same variant (OR: 0.22, *p* = 0.028). Considering the different outcomes of severity 3 as a common outcome, history of vaccination was evaluated as a statistically significant protective factor (OR = 0.19, *p* = 0.015) (Table S4). The rest of the results of this multivariate analysis are presented in Table S4. Restricting the multivariate analysis in unvaccinated adults, age, male sex, respiratory system disorders, malignancies, and metabolic disorders/endocrinopathies were associated with poorer prognosis (Table S5).

**Figure 4:**
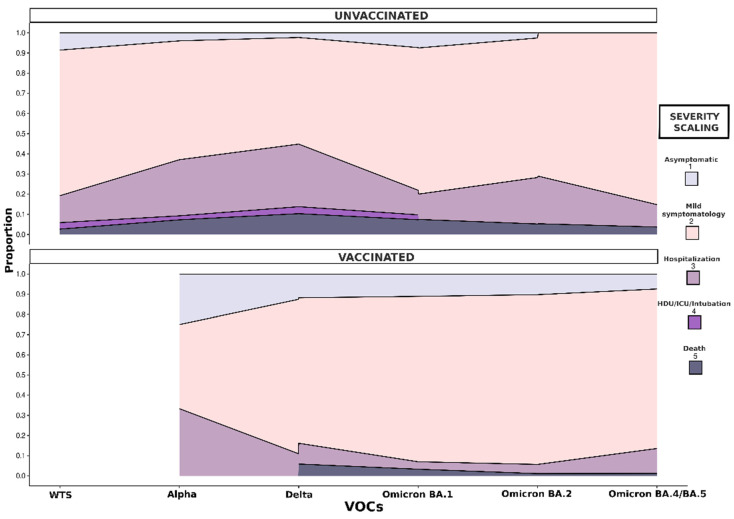
Comparison of COVID-19 severity among vaccinated and unvaccinated participants.

Figure 4 illustrates the morbidity and variation of COVID-19 severity by VOC among unvaccinated and vaccinated participants. Immunization via vaccines against COVID-19 appeared to reduce severity. Hospitalization, HDU/ICU admission, intubation, and death caused by SARS-CoV-2 infection were more commonly recorded among unvaccinated individuals, while the possibility of experiencing mild symptomology was higher in vaccinated individuals.

## 4. Discussion

In this study, we analyzed data collected from 913 COVID-19 cases in a region of central Greece between October 2020 and July 2022. Clinical manifestations and outcomes among participants infected by different SARS-CoV-2 VOCs were compared. We observed that in adults and children, symptom profiles differed based on etiologic variants.

The majority of pediatric COVID-19 patients developed mild symptoms, with fewer than 20% remaining completely asymptomatic. A similar proportion has been reported in another study, in which 25% of children did not experience symptoms [9]. In our study, children infected by Omicron variants developed clinical manifestations more frequently compared to those infected by the non-Omicron variant: Omicron: 86.4% and non-Omicron: 73.5%. However, the Delta variant was found to cause a similar proportion of symptomatic cases (86.7%) as Omicron variants (86.4%), followed by Alpha: 75.0% and WTS: 54.5%. Similar results were reported in another study, where among children seen in an emergency department of a Canadian hospital, 92.7% of those infected with Omicron and 91.6% with Delta developed symptoms, while the proportion of symptomatic children infected by other VOCs were less than 90% (WTS: 86.6%, Alpha: 82.3%) [10].

In our study, the majority of adults developed symptoms, with only 6% declared completely asymptomatic. No statistically significant differences were found in the proportions of asymptomatic cases among VOCs. A high heterogeneity in the proportion of asymptomatic cases is observed among studies [11,12]. A meta-analysis found that the pooled percentage of asymptomatic cases was 40.50% (95% CI: 33.5–47.5%; I2 = 99%; *p* < 0.001) [12]. The percentage of asymptomatic ranged from 3.7% to 87.5%. The proportion of asymptomatic individuals in our study was estimated among the lowest from those reported in the aforementioned literature. This could be attributed to the fact that our sample was drawn from cases admitted to the hospital emergency department. It is also worth noting that the NPHO organized daily COVID-19 surveillance points. Therefore, a proportion of asymptomatic individuals may have been diagnosed at these facilities as they did not require medical assistance.

Considering that the majority of COVID-19 cases experienced symptoms, their duration could be further studied. Non-Omicron VOCs more frequently caused clinical presentations exceeding 20 days, with the Delta variant being prevalent (9.9%), followed by Alpha (9.6%) and WTS (4.5%). The duration of acute infection for each specific variant has not been extensively studied or reported in a systematic manner.

Fever and cough were the most common symptoms among Delta and Omicron infections in children; fever was reported by 80.0% and cough by 60.0% of Delta cases, whereas the corresponding percentages were 62.1% and 39.4% in Omicron cases. Similar findings have been reported in other studies [10,13,14]. In adults, fever was the most frequently self-reported symptom in all waves, ranging from 81.8% during the WTS wave to 64.8% during the Omicron BA.1 wave. Flisiak R et al. also recorded a steady decline in fever until the predominance of the Omicron variant [15]. Cough was recorded at high rates throughout all stages of our study. This observation is consistent with findings from a study by DeWitt et al., who also noted cough as the most frequently reported symptom [16].

Omicron cases experienced symptoms of the upper respiratory tract more frequently. In particular, 28.8% of children with Omicron infection experienced sore throat, and 47.0% experienced nasal congestion/discharge, while 41.0% of adults infected by the same variant developed sore throat and 49.6% nasal congestion/discharge. Similar results have been reported in other studies [17,18]. Menni et al. observed that sore throat and hoarse voice were more prevalent among Omicron cases compared to Delta infections [18]. This finding may be attributed to mutations primarily occurring in the Spike protein, which enhance the virus’s affinity for binding to receptors in the upper respiratory system and decrease the tissue tropism of Omicron in the lungs compared to previous strains [19,20,21].

During the Omicron wave, a decrease in the incidence of taste and smell disorders was observed. The incidence of taste disorders ranged from 44.9% during the WTS wave to 13.3% during the Omicron wave, while the incidence of smell disorders ranged from 39.8% during the WTS wave to 10.9% during the Omicron wave. This finding is supported by another study [16]. During the initial phase of the pandemic, VOCs caused smell and taste disorders more often (55% during the pre-Delta period). However, there was a decline during the Delta phase and a further decrease during the Omicron VOCs (17%) [16]. Similar to findings by Coelho et al., olfactory and gustatory dysfunctions were more common in patients with the Alpha variant (64%), followed by Delta (57%), and they were less frequent in Omicron (21%); however, this study did not include patients infected with WTS [22]. Data from three prospective household cohorts comparing SARS-CoV-2 symptomatology of WTS/Alpha to Omicron BA.1/BA.2 variants are in line with our results; Omicron infections were associated with lower odds of loss of smell or taste (OR: 0.14) [23].

Among children, GI symptoms more frequently developed in cases of Omicron (10.6%), but the difference was not calculated as statistically significant among VOCs (*p* = 0.662). Similar results have been found in other studies [24,25]. A published article reported that SARS-CoV-2 RNA was more frequently present in anal swabs of Omicron cases compared to cases infected with previous variants, a finding that may support our results [26]. Conversely, symptoms of the GI tract among adults were self-reported more commonly in the Alpha variant (31.4%), followed by Omicron BA.4/BA.5 (28.4%). Another study focusing on the same age group claims that pre-Delta variants more commonly resulted in manifestations from the GI tract than the Delta and Omicron variants (pre-Delta: 44.9%, Delta: 35.6%, and Omicron: 30.1%, *p* < 0.001) [27].

The frequency of GI symptoms reported in the literature vary by VOC; several possible pathophysiological explanations exist [28]. Molecular perspectives support that the high ACE-2 protein expression found in intestinal epithelial cells may facilitate the entry of SARS-CoV-2 into host cells [29]. Thus, mutations may change virus tropism, as already described. Alteration of gut microbiota may be another reason for GI symptoms [30,31]. Since the onset of the pandemic, a variety of drugs have been utilized, including antibiotics and antivirals, potentially interfering with the GI microbiota [32].

Given the lifting or easing of public and personal health measures during the predominance of the Omicron variant, the higher proportion of upper respiratory tract infections or GI symptoms could be caused by other viral or bacterial co-infections. Additionally, a “test all” approach occurred during the Omicron period, as rapid antigen tests were available, compared to the non-Omicron period, characterized by a more selective strategy testing individuals who were unwell or experienced core COVID-19 symptoms.

During our study period, we found that Alpha or Delta cases had a higher risk for hospitalization, diagnosis of pneumonia, and death compared to cases infected by the WTS or Omicron variants (during the Alpha wave, we observed hospitalizations at 37.2%, diagnosis of pneumonia at 25.0%, and death at 7.1%, and during the Delta wave, hospitalizations at 35.9%, diagnosis of pneumonia at 28.9%, and death at 9.2%; while during the WTS wave, we observed hospitalizations at 20.5%, diagnosis of pneumonia at 16.5%, and death at 2.8%, and during Omicron, hospitalizations at 8.0%, diagnosis of pneumonia at 4.1%, and death at 1.8%). Our findings are consistent with other studies [33,34,35]. Several factors should be considered as contributing to this outcome. 

The Omicron variant’s intrinsic virologic properties could explain its lower severity. Although the Omicron variant has demonstrated partial vaccine escape and higher transmissibility, it presents lower pathogenicity and a lower replication rate in lung tissues [21,33]. This may be due to mutations near the furin cleavage site, such as S655Y, or the combination of mutations S477N, Q498R, and N501Y in the Omicron variants’ Spike protein, as these increase the binding affinity for the ACE-2 receptor [36,37]. Likely owing to the tropism of Omicron, pro-inflammatory cytokines and chemokines were found lower in the lungs of Omicron-infected mice than in those infected by previous variants, further explaining the lower severity in cases of Omicron [38]. During the genetic makeup of the virus, it has also been reported that Omicron proved less fusogenic than the Delta and WTS since the Spike protein of Omicron is less efficiently cleaved into two subunits compared to the two other variants [39,40]. This could further explain the lower pathogenicity of Omicron and subsequently the lower frequency of pneumonia diagnosis, respiratory failure/syndromes, hospitalizations, ICU admissions, intubations, and deaths [41,42].

Despite the variants’ characteristics, it should be considered that during the predominance of the Omicron variant, a greater number of individuals were immunized via vaccination or through SARS-CoV-2 infection, either one or multiple times. In our study, vaccination coverage was over 80% during the Omicron period, and the proportion of vaccinated participants with three doses was nearly 70% during the last pandemic wave. Booster shots have proven effective in reducing the risk of severe disease and death, with the protection afforded following a booster unaffected by the initial vaccination [33,34,43]. Moreover, previous SARS-CoV-2 infection was protective, especially in unvaccinated populations [34]. During Omicron predominance, one in ten of our participants mentioned a history of COVID-19, while no participants reported SARS-CoV-2 history in pre-Omicron waves.

We found that Delta and Alpha variants displayed the highest mortality (Delta: 9.2%, Alpha: 7.1%). In other studies, Delta also presented with the highest case fatality rate, followed by Alpha [44,45,46,47]. Despite the high virulence of Delta, factors including healthcare worker (HCW) burnout syndrome and exceeding capacities of hospitals/ICUs during the Delta wave should be considered. The lowest mortality was calculated in Omicron variants (1.8%); these results are similar to the outcomes of other studies [42,48,49]. As the pandemic progressed, lower pathogenicity of the predominant variant, the experience of HCWs, health unit staffing, and enhanced ICU capacity, as well as immunity from vaccinations and previous infection, could have resulted in lower COVID-19 severity. As the pandemic threatened global public health, strict compliance with public health guidelines may have inhibited other co-infections, which would have worsened clinical outcomes.

## 5. Limitations

Our study presents a number of limitations. Firstly, study participants are not representative of the general population since they were primarily sourced from patients visiting the hospital emergency department; this could explain the higher proportion of symptomatic cases. Secondly, excluding hospitalized cases whose data were drawn from medical records, the prevalence of symptoms in non-hospitalized cases was self-reported. Moreover, strains from the Beta and Gamma variants were not included in this study, as there were not enough samples in our region. Another limitation is the continuous downward trend in participants’ response rates during the study period, resulting in a varied number of cases among each variant group. During BA.4/BA.5 predominance, three months following the start of BA.4/BA.5 circulation, our laboratory did not receive samples for molecular analysis; this further explains the low number of participants in this group. Finally, NGS was not conducted on all samples, with the remaining samples characterized within the same period and the S gene dropout.

However, our study has several advantages. The majority of VOCs are included and compared simultaneously. Furthermore, the inclusion of all age groups provides a macroscopic overview of the clinical characteristics and outcomes of each variant. Our findings and their interpretations are enhanced, as the vaccination initiation period coincides with our study period.

## 6. Conclusions

Clinical manifestations and COVID-19 outcomes appeared to differ during each VOC-predominant period. As the pandemic progressed, the proportion of severe cases decreased, while mild/moderate cases increased. This could be attributed to the characteristics of the virus, the host, the virus–host interaction, and the effect of environmental factors. In our study, vaccination lessened severe COVID-19 outcomes. At present, vaccination could be one of the most effective and feasible approaches to prevent adverse health impacts. Despite the fact that current circulating VOCs are less virulent, the COVID-19 vaccine continues to play a protective role for severe outcomes.

## Figures and Tables

**Table 1:** Overview of symptoms of SARS-CoV-2 ^1^-positive children (<18 years old) among VOCs ^2^.

Participants <18 Years Old	Total	Non-Omicron Variants (Total)	Non-Omicron Variants			Omicron Variants (Total)	Omicron Variants			*p*-Value
			WTS ^3^	Alpha	Delta		Omicron BA.1	Omicron BA.2	Omicron BA.4/BA.5	
	(N = 100)	(N = 34)	(N = 11)	(N = 8)	(N = 15)	(N = 66)	(N = 30)	(N = 16)	(N = 20)	
Severity										
0	18 (18.0%)	9 (26.5%)	5 (45.5%)	2 (25.0%)	2 (13.3%)	9 (13.6%)	7 (23.3%)	1 (6.3%)	1 (5.0%)	N/A ^4^
1	75 (75.0%)	22 (64.7%)	6 (54.5%)	4 (50.0%)	12 (80.0%)	53 80.3%)	23 (76.7%)	13 (81.3%)	17 (85.0%)	
2	7 (7.0%)	3 (8.8%)	0 (0%)	2 (25.0%)	1 (6.7%)	4 (6.1%)	0 (0%)	2 (12.5%)	2 (10.0%)	
3	0 (0%)	0 (0%)	0 (0%)	0 (0%)	0 (0%)	0 (0%)	0 (0%)	0 (0%)	0 (0%)	
Symptoms	82 (82.0%)	25 (73.5%)	6 (54.5%)	6 (75.0%)	13 (86.7%)	57 (86.4%)	23 (76.7%)	15 (93.8%)	19 (95.0%)	0.039
Duration of symptoms (days)	2.00 (2.00)	2.5 (5.5)	2.00 (0)	2.00 (0)	5.00 (4.00)	2.00 (2.00)	2.00 (2.00)	2.00 (1.00)	3.00 (2.00)	0.060
Cough	36 (36.0%)	10 (29.4%)	0 (0%)	1 (12.5%)	9 (60.0%)	26 (39.4%)	8 (26.7%)	8 (50.0%)	10 (50.0%)	0.007
Duration of cough (days)	4.00 (4.25)	5.5 (4.75)	-	10.0 (0)	4.00 (5.00)	4.00 (2.75)	3.00 (2.50)	3.50 (1.00)	6.50 (3.00)	0.105
Fever	62 (62.0%)	21 (61.8%)	5 (45.5%)	4 (50.0%)	12 (80.0%)	41 (62.1%)	14 (46.7%)	14 (87.5%)	13 (65.0%)	0.046
Maximum value (°C)	38.25 (0.68)	38.00 (0.25)	38.2 (0.500)	38.0 (0.150)	38.0 (0.825)	38.50 (0.70)	38.2 (0.500)	38.6 (0.875)	38.5 (0.600)	0.626
Duration of fever (days)	2.00 (1.00)	2.00 (1.00)	2.00 (0)	2.00 (1.50)	3.00 (3.00)	2.00 (1.00)	1.50 (1.00)	2.00 (2.00)	2.00 (1.00)	0.020
Sore throat (pharyngula)	21 (21.0%)	2 (5.9%)	0 (0%)	0 (0%)	2 (13.3%)	19 (28.8%)	9 (30.0%)	2 (12.5%)	8 (40.0%)	0.034
Nasal congestion/discharge	37 (37.0%)	6 (17.6%)	1 (9.1%)	1 (12.5%)	4 (26.7%)	31 (47.0%)	13 (43.3%)	7 (43.8%)	11 (55.0%)	0.074
Duration of nasal congestion (days)	3.00 (3.00)	2.00 (2.25)	5.00 (0)	2.00 (0)	2.00 (1.25)	4.00 (3.00)	2.00 (3.00)	4.00 (1.50)	4.00 (3.00)	0.247
Headache	24 (24.0%)	5 (14.7%)	1 (9.1%)	0 (0%)	4 (26.7%)	19 (28.8%)	8 (26.7%)	6 (37.5%)	5 (25.0%)	0.342
Headache intensity										
Severe	10 (10.0%)	5 (14.7%)	1 (9.1%)	0 (0%)	4 (26.7%)	5 (76%)	2 (6.7%)	2 (12.5%)	1 (5.0%)	0.144
Mild	14 (14.0%)	0 (0.0%)	0 (0%)	0 (0%)	0 (0%)	14 (21.2%)	6 (20.0%)	4 (25.0%)	4 (20.0%)	
Fatigue	30 (30.0%)	7 (20.6%)	3 (27.3%)	0 (0%)	4 (26.7%)	23 (34.8%)	13 (43.3%)	5 (31.3%)	5 (25.0%)	0.275
Taste disorders	11 (11.0%)	7 (20.6%)	1 (9.1%)	1 (12.5%)	5 (33.3%)	4 (6.1%)	2 (6.7%)	2 (12.5%)	0 (0%)	0.056
Duration of taste disorders	4.00 (4.00)	7.00 (5.50)	2.00 (0)	4.00 (0)	7.00 (3.00)	3.50 (1.00)	3.00 (0)	4.00 (0)	-	0.383
Smell disorders	9 (9.0%)	7 (20.6%)	2 (18.2%)	0 (0%)	5 (33.3%)	2 (3.0%)	1 (3.3%)	1 (6.3%)	0 (0%)	0.007
Duration of smell disorders (days)	7.00 (8.00)	7.00 (7.50)	16.0 (14.0)	-	7.00 (3.00)	2.00 (1.00)	1.00 (0)	3.00 (0)	-	0.525
GI ^5^ symptoms	8 (8.0%)	1 (2.9%)	0 (0%)	0 (0%)	1 (6.7%)	7 (10.6%)	3 (10.0%)	1 (6.3%)	3 (15.0%)	0.662
Appetite disorders										
No	96 (96.0%)	32 (94.1%)	11 (100%)	7 (87.5%)	14 (93.3%)	64 (97.0%)	30 (100%)	16 (100%)	18 (90.0%)	0.371
Increased	2 (2.0%)	1 (2.9%)	0 (0%)	1 (12.5%)	0 (0%)	1 (1.5%)	0 (0%)	0 (0%)	1 (5.0%)	
Decreased	2 (2.0%)	1 (2.9%)	0 (0%)	0 (0%)	1 (6.7%)	1 (1.5%)	0 (0%)	0 (0%)	1 (5.0%)	
Dyspnea	1	0 (0.0%)	0 (0%)	0 (0%)	0 (0%)	1 (1.5%)	0 (0%)	0 (0%)	1 (5.0%)	0.544

^1^ SARS-CoV-2: severe acute respiratory syndrome coronavirus 2; ^2^ VOCs: variants of concern; ^3^ WTS: wild type strain; ^4^ N/A: not applicable; ^5^ GI: gastrointestinal.

**Table 2:** Overview of symptoms of SARS-CoV-2 ^1^-positive adult (≥18 age years) participants among VOCs ^2^.

Participants ≥18 Years Old	Total	Non-Omicron Variants (Total)	Non-Omicron Variants			Omicron Variants (Total)	Omicron Variants			*p*-Value
			WTS ^3^	Alpha	Delta		Omicron BA.1	Omicron BA.2	Omicron BA.4/BA.5	
	(N = 813)	(N = 474)	(N = 176)	(N = 156)	(N = 142)	(N = 339)	(N = 142)	(N = 109)	(N = 88)	
Severity										
0	51 (6.3%)	26 (5.5%)	11 (6.3%)	7 (4.5%)	8 (5.6%)	25 (7.4%)	11 (7.7%)	9 (8.3%)	5 (5.7%)	<0.001
1	577 (71.0%)	302 (63.7%)	129 (73.3%)	90 (57.7%)	83 (58.5%)	275 (81.1%)	117 (82.4%)	88 (80.7%)	70 (79.5%)	
2	148 (18.2%)	115 (24.3%)	30 (17.0%)	47 (30.1%)	38 (26.8%)	33 (9.7%)	11 (7.7%)	11 (10.1%)	11 (12.5%)	
3	37 (4.6%)	31 (6.5%)	6 (3.4%)	12 (7.7%)	13 (9.2%)	6 (1.8%)	3 (2.1%)	1 (0.9%)	2 (2.3%)	
Symptoms	762 (93.7%)	448 (94.5%)	165 (93.8%)	149 (95.5%)	134 (94.4%)	314 (92.6%)	131 (92.3%)	100 (91.7%)	83 (94.3%)	0.813
Duration of symptoms (days)	5.00 (7.00)	7.00 (7.00)	6.00 (7.00)	10.0 (10.0)	7.00 (6.75)	5.00 (3.00)	5.00 (4.00)	5.00 (3.00)	4.00 (3.00)	<0.001
Cough	328 (40.3%)	184 (38.8%)	70 (39.8%)	58 (37.2%)	56 (39.4%)	144 (42.5%)	58 (40.8%)	47 (43.1%)	39 (44.3%)	0.894
Duration of cough (days)	7.00 (6.00)	7.00 (5.00)	7.00 (8.75)	7.00 (5.75)	7.00 (5.00)	6.00 (6.00)	7.00 (5.00)	5.00 (4.50)	6.00 (8.00)	0.187
Fever	607 (74.7%)	381 (80.4%)	144 (81.8%)	124 (79.5%)	113 (79.6%)	226 (66.7%)	92 (64.8%)	71 (65.1%)	63 (71.6%)	<0.001
Maximum value (°C)	38.0 (0.80)	38.0 (0.50)	38.0 (0.80)	38.0 (0.70)	38.3 (0.80)	38.0 (1.05)	38.0 (0.90)	38.0 (1.00)	38.5 (1.00)	<0.001
Duration of fever (days)	3.00 (2.00)	3.00 (4.00)	3.00 (4.25)	3.00 (4.00)	3.00 (3.00)	2.00 (1.00)	2.00 (1.25)	2.00 (2.00)	2.00 (1.00)	<0.001
Sore throat (pharyngula	237 (29.2%)	98 (20.7%)	42 (23.9%)	29 (18.6%)	27 (19.0%)	139 (41.0%)	64 (45.1%)	40 (36.7%)	35 (39.8%)	<0.001
Nasal congestion/discharge	269 (33.1%)	101 (21.3%)	42 (23.9%)	29 (18.6%)	30 (21.1%)	168 (49.6%)	72 (50.7%)	55 (50.5%)	41 (46.6%)	<0.001
Duration of nasal congestion(days)	5.00 (4.00)	5.00 (5.00)	5.00 (3.00)	4.00 (7.00)	5.00 (7.00)	5.00 (3.00)	5.00 (3.00)	5.00 (4.00)	5.00 (3.00)	0.211
Headache	337 (41.5%)	179 (37.8%)	89 (50.6%)	49 (31.4%)	41 (28.9%)	158 (46.6%)	65 (45.8%)	45 (41.3%)	48 (54.5%)	<0.001
Headache intensity										
Severe	253 (31.1%)	150 (31.6%)	74 (42.0%)	38 (24.4%)	38 (26.8%)	103 (30.4%)	39 (27.5%)	25 (22.9%)	39 (44.3%)	<0.001
Mild	84 (10.3%)	29 (6.1%)	15 (8.5%)	11 (7.1%)	3 (2.1%)	55 (16.2%)	26 (18.3%)	20 (18.3%)	9 (10.2%)	
Fatigue	466 (57.3%)	255 (53.8%)	98 (55.7%)	83 (53.2%)	74 (52.1%)	211 (62.2%)	75 (52.8%)	68 (62.4%)	68 (77.3%)	0.002
Taste disorders	209 (25.7%)	164 (34.6%)	79 (44.9%)	48 (30.8%)	37 (26.1%)	45 (13.3%)	18 (12.7%)	11 (10.1%)	16 (18.2%)	<0.001
Duration of taste disorders (days)	7.00 (9.00)	8.00 (7.00)	7.00 (3.75)	7.00 (7.00)	12.5 (9.25)	5.00 (7.00)	5.00 (5.25)	4.00 (3.00)	5.00 (12.3)	<0.001
Smell disorders	183 (22.5%)	146 (30.8%)	70 (39.8%)	39 (25.0%)	37 (26.1%)	37 (10.9%)	17 (12.0%)	8 (7.3%)	12 (13.6%)	<0.001
Duration of smell disorders (days)	7.00 (8.00)	7.00 (7.25)	7.00 (4.00)	7.00 (6.00)	12.5 (9.25)	5.00 (7.00)	5.00 (3.00)	4.50 (6.25)	4.50 (8.75)	<0.001
GI ^4^ symptoms	152 (18.7%)	96 (20.3%)	35 (19.9%)	49 (31.4%)	12 (8.5%)	56 (16.5%)	20 (14.1%)	11 (10.1%)	25 (28.4%)	<0.001
Appetite disorders										
No	746 (91.8%)	433 (91.4%)	169 (96.0%)	125 (80.1%)	139 (97.9%)	313 (92.3%)	135 (95.1%)	102 (93.6%)	76 (86.4%)	<0.001
Increased	11 (1.4%)	7 (1.5%)	1 (0.6%)	6 (3.8%)	0 (0%)	4 (1.2%)	3 (2.1%)	1 (0.9%)	0 (0%)	
Decreased	56 (6.9%)	34 (7.2%)	6 (3.4%)	25 (16.0%)	3 (2.1%)	22 (6.5%)	4 (2.8%)	6 (5.5%)	12 (13.6%)	
Dyspnea	138 (17.0%)	111 (23.4%)	35 (19.9%)	37 (23.7%)	39 (27.5%)	27 (8.0%)	12 (8.5%)	6 (5.5%)	9 (10.2%)	<0.001

^1^ SARS-CoV-2: severe acute respiratory syndrome coronavirus 2; ^2^ VOCs: variants of concern; ^3^ WTS: wild type strain; ^4^ GI: gastrointestinal.

**Table 3:** Overview of disease outcomes of SARS-CoV-2 ^1^-positive adult (≥18 age years) participants among VOCs ^2^.

Participants ≥18 Years Old	Total	Non-Omicron Variants (Total)	Non-Omicron Variants			Omicron Variants (Total)	Omicron Variants			*p*-Value
			WTS ^3^	Alpha	Delta		BA.1	BA.2	BA.4/BA.5	
	813	474	(N = 176)	(N = 156)	(N = 142)	339	(N = 142)	(N = 109)	(N = 88)	
Hospitalization because of COVID-19 ^4^	172 (21.2%)	145 (30.6%)	36 (20.5%)	58 (37.2%)	51 (35.9%)	27 (8.0%)	14 (9.9%)	12 (11.0%)	13 (14.8%)	<0.001
Duration of hospitalization (days)	6.00 (6.00)	6.00 (6.00)	7.00 (5.75)	6.00 (7.50)	6.00 (5.00)	5.00 (5.00)	6.50 (8.25)	4.00 (2.25)	3.00 (2.00)	0.084
Diagnosis of pneumonia	123 (15.1%)	109 (23.0%)	29 (16.5%)	39 (25.0%)	41 (28.9%)	14 (4.1%)	9 (6.3%)	3 (2.8%)	2 (2.3%)	<0.001
HDU ^5^	15 (1.5%)	13 (2.7%)	5 (2.8%)	3 (1.9%)	5 (3.5%)	2 (0.6%)	1 (0.7%)	1 (0.9%)	0 (0%)	0.272
Duration in HDU (days)	9.00 (5.00)	9.00 (3.00)	6.00 (3.00)	9.00 (4.00)	9.00 (6.00)	27.5 (12.50)	40.0 (0)	15.0 (0)	-	0.255
Hospitalization in ICU ^6^	16 (2.0%)	15 (3.2%)	5 (2.8%)	4 (2.6%)	6 (4.2%)	1 (0.3%)	1 (0.7%)	0 (0%)	0 (0%)	0.079
Duration in ICU (days)	11.50 (14.75)	10.00 (14.00)	10.0 (4.00)	5.50 (8.00)	21.0 (6.00)	36.0 (0)	36.0 (0)	-	-	0.247
Intubation	17 (2.1%)	15 (3.2%)	5 (2.8%)	4 (2.6%)	6 (4.2%)	2 (0.6%)	1 (0.7%)	1 (0.9%)	0 (0%)	0.170
Duration of intubation (days)	9.00 (7.00)	9.00 (6.50)	9.00 (4.00)	7.50 (5.50)	9.00 (8.75)	11.50 (1.50)	10.0 (0)	13.0 (0)	-	0.835
Death	35 (4.3%)	29 (6.1%)	5 (2.8%)	11 (7.1%)	13 (9.2%)	6 (1.8%)	3 (2.1%)	1 (0.9%)	2 (2.3%)	0.004

^1^ SARS-CoV-2: severe acute respiratory syndrome coronavirus 2; ^2^ VOCs: variants of concern; ^3^ WTS: wild type strain; ^4^ COVID-19: coronavirus disease 2019; ^5^ HDU: high-dependency care unit; ^6^ ICU: intensive care unit.

## Data Availability

The data are not publicly available as they contain sensitive information at the individual level.

## Supplementary Materials

The following supporting information is available at https://www.mdpi.com/article/10.3390/microorganisms12122573/s1, Table S1: Demographic characteristics of SARS-CoV-2-positive children (<18 years old) participants; Table S2: Demographic characteristics of SARS-CoV-2-positive adult (≥18 age years) participants; Table S3: Multivariate analysis of hospitalization, pneumonia, ICU admission, intubation, and death in Omicron and non-Omicron variants among adult participants; Table S4: Multivariate analysis of severe COVID-19 (ICU admission, intubation, and death together) in non-Omicron variants among adult participants; Table S5: Multivariate analysis of hospitalization and pneumonia among unvaccinated adult participants; Figure S1: The proportion and the course of symptoms per VOC due to SARS-CoV-2 infection.
