# Supplementary material for: The Clinical Anatomy of SARS-CoV-2 Variants of Concern in Central Greece During October 2020–July 2022
Source: Microorganisms. 2024 Dec 13;12(12):2573. doi: 10.3390/microorganisms12122573 (PMC11678192; doi:10.3390/microorganisms12122573)
Supplement: Supplementary file 1 [file microorganisms-12-02573-s001.zip › microorganisms-3298404-supplementary.pdf]

Table S1. Demographic characteristics of SARS-CoV-2[1] positive children (<18 years old) participants.

**Participants <18 age years old**	**Total**	**Non-Omicron variants**			**Omicron variants**			**P-value**
		**WTS[2]**	**Alpha**	**Delta**	**Omicron BA.1**	**Omicron BA.2**	**Omicron BA.4/BA.5**	
	**(N=100)**	**(N=11)**	**(N=8)**	**(N=15)**	**(N=30)**	**(N=16)**	**(N=20)**	
Sex								
Male	60 (60%)	8 (72.7%)	4 (50.0%)	6 (40.0%)	24 (80.0%)	9 (56.2%)	9 (45.0%)	0.061
Female	40 (40%)	3 (27.3%)	4 (50.0%)	9 (60.0%)	6 (20.0%)	7 (43.8%)	11 (55.0%)	
Age (years)	11.0 (5.00)	12.0 (3.00)	12.0 (6.75)	11.0 (5.00)	10.0 (3.75)	11.5 (8.75)	9.00 (9.00)	0.802
Commorbidity	8 (8%)	0 (0.0%)	0 (0.0%)	3 (20.0%)	2 (6.7%)	1 (6.2%)	2 (10.0%)	0.436
Vaccination	28 (28%)	0 (0.0%)	0 (0.0%)	0 (0.0%)	17 (56.7%)	4 (25.0%)	7 (35.0%)	**<0.001**
Doses								
0	72 (72%)	11 (100.0%)	8 (100.0%)	15 (100.0%)	13 (43.3%)	12 (75.0%)	13 (65.0%)	N/A[3]
1	8 (8%)	0 (0.0%)	0 (0.0%)	0 (0.0%)	8 (26.7%)	0 (0.0%)	0 (0.0%)	
2	20 (20%)	0 (0.0%)	0 (0.0%)	0 (0.0%)	9 (30.0%)	4 (25.0%)	7 (35.0%)	
3	0 (0%)	0 (0.0%)	0 (0.0%)	0 (0.0%)	0 (0.0%)	0 (0.0%)	0 (0.0%)	
Previous SARS-CoV-2 infection	7 (7%)	0 (0.0%)	0 (0.0%)	0 (0.0%)	2 (6.7%)	0 (0.0%)	5 (25.0%)	**0.018**

[1] SARS-CoV-2: severe acute respiratory syndrome coronavirus 2; [2] WTS: wild type strain; [3] N/A: not applicable.

Table S2: Demographic characteristics of SARS-CoV-2[1] positive adult (≥18 age years) participants.

**Participants ≥18 age years old**	**Total**	**Non-Omicron variants**			**Omicron variants**			**P-value**
		**WTS[2]**	**Alpha**	**Delta**	**Omicron BA.1**	**Omicron BA.2**	**Omicron BA.4/BA.5**	
	**(N=813)**	**(N=176)**	**(N=156)**	**(N=142)**	**(N=142)**	**(N=109)**	**(N=88)**	
Sex								
Male	349 (42.9%)	89 (50.6%)	77 (49.4%)	63 (44.4%)	47 (33.1%)	38 (34.9%)	35 (39.8%)	**0.007**
Female	464 (57.1%)	87 (49.4%)	79 (50.6%)	79 (55.6%)	95 (66.9%)	71 (65.1%)	53 (60.2%)	
Age (years)	47.0 (25.0)	44.5 (27.3)	53.0 (22.3)	47.0 (38.5)	41.0 (22.0)	48.0 (20.0)	48.0 (23.3)	**<0.001**
Commorbidity	387 (47.6%)	84 (47.7%)	102 (65.4%)	69 (48.6%)	47 (33.1%)	43 (39.4%)	42 (47.7%)	**<0.001**
Vaccination	354 (43.5%)	0 (0.0%)	12 (7.7%)	70 (49.3%)	114 (80.3%)	84 (77.1%)	74 (84.1%)	**<0.001**
Doses								
0	459 (56.5%)	176 (100.0%)	144 (92.3%)	72 (50.7%)	28 (19.7%)	25 (22.9%)	14 (15.9%)	**<0.001**
1	34 (4.2%)	0 (0.0%)	9 (5.8%)	8 (5.6%)	12 (8.5%)	4 (3.7%)	1 (1.1%)	
2	122 (15.0%)	0 (0.0%)	3 (1.9%)	59 (41.5%)	30 (21.1%)	16 (14.7%)	14 (15.9%)	
3	198 (24.4%)	0 (0.0%)	0 (0.0%)	3 (2.1%)	72 (50.7%)	64 (58.7%)	59 (67.0%)	
Previous SARS-CoV-2 infection	37 (4.6%)	0 (0.0%)	0 (0.0%)	0 (0.0%)	14 (9.9%)	12 (11.0%)	11 (12.5%)	**<0.001**
ORF[3] gene (Ct[4])	18.0 (6.00)	20.0 (6.25)	18.0 (6.00)	16.0 (7.00)	20.0 (7.00)	17.0 (6.00)	18.0 (7.00)	**<0.001**

[1] SARS-CoV-2: severe acute respiratory syndrome coronavirus 2; [2] WTS: wild type strain; [3] ORF: Open Reading Frames; [4] Ct: cycle threshold.

Table S3. Multivariate analysis of hospitalization, pneumonia, ICU[1] admission, intubation and death in Omicron and non-Omicron variants among adult participants.

	Hospitalization						Pneumonia						ICU			Intubation			Death		
	**Non-Omicron (N=474)**			**Omicron (N=339)**			**Non-Omicron (N=474)**			**Omicron (N=339)**			**Non-Omicron (N=474)**			**Non-Omicron (N=474)**			**Non-Omicron (N=474)**		
	**OR[2]**	**95% C.I.[3]**	**P-value**	**OR**	**95% C.I.**	**P-value**	**OR**	**95% C.I.**	**P-value**	**OR**	**95% C.I.**	**P-value**	**OR**	**95% C.I.**	**P-value**	**OR**	**95% C.I.**	**P-value**	**OR**	**95% C.I.**	**P-value**
Age	1.06	1.05 – 1.09	<0.001	1.14	1.09 – 1.21	**<0.001**	1.06	1.04 – 1.08	<0.001	1.08	1.03 – 1.15	**0.006**	1.04	1.00 – 1.09	0.054	1.05	1.00 – 1.10	0.038	1.07	1.04 – 1.12	<0.001
Sex (M/F)	1.68	1.01 – 2.81	0.047	1.89	0.62 – 5.99	0.265	1.67	0.98 – 2.88	0.059	4.66	0.82 – 41.19	0.109	3.34	0.95 – 14.09	0.074	3.56	0.97 – 15.64	0.067	3.24	1.17 – 9.73	0.028
Autoimmune/ autoinflammatory diseases	8.56	2.36 – 32.44	0.001	0.67	0.12 – 2.85	0.607	2.32	0.57 – 8.31	0.209	0.28	0.01 – 2.60	0.333	2.14	0.09 – 18.82	0.551	-	-	0.991	3.06	0.24 – 25.18	0.338
Dementia/Insomnia/ Neurological disorders	0.88	0.41 – 1.88	0.749	9.07	1.57 – 57.28	**0.015**	1.01	0.48 – 2.09	0.971	0.71	0.03 – 8.60	0.801	0.64	0.11 – 2.68	0.573	0.66	0.12 – 2.72	0.593	1.77	0.61 – 4.90	0.280
Respiratory system disorders	2.07	0.75 – 5.76	0.156	0.98	0.12 – 6.41	0.980	3.46	1.33 – 9.14	0.011	3.19	0.20 – 39.26	0.373	0.64	0.07 – 3.71	0.655	1.04	0.15 – 5.26	0.968	1.05	0.20 – 4.32	0.950
Malignancies	18.93	2.68 – 392.66	0.012	7.49	1.29 – 46.48	**0.026**	2.45	0.54 – 12.27	0.252	0.54	0.01 – 8.97	0.702	1.96	0.09 – 15.83	0.580	1.52	0.07 – 12.18	0.733	0.41	0.02 – 3.16	0.463
Hematological disorders	2.75	0.64 – 11.31	0.160	21.49	1.11 – 412.54	**0.046**	1.56	0.35 – 6.11	0.539	9.16	0.25 – 278.15	0.185	1.58	0.06 – 13.13	0.719	3.33	0.32 – 22.22	0.251	2.40	0.32 – 14.07	0.357
GI[4] system disorders	0.66	0.13 – 2.92	0.588	0.69	0.00 – 101.13	0.897	1.31	0.30 – 5.28	0.709	-	-	0.999	2.59	0.12 – 20.85	0.430	2.63	0.12 – 21.54	0.424	6.95	1.11 – 39.09	0.029
Immunosuppression/Pregnancy	1.86	0.07 – 21.33	0.649	-	-	0.993	-	-	0.984	-	-	0.999	-	-	0.991	-	-	0.996	-	-	0.991
Cardiovascular diseases	0.86	0.47 – 1.57	0.628	0.59	0.13 – 2.45	0.473	0.84	0.44 – 1.58	0.588	5.54	0.76 – 59.10	0.115	0.81	0.16 – 4.21	0.794	1.01	0.19 – 6.21	0.992	1.64	0.44 – 6.98	0.476
Metabolic disorders/ endocrinopathies	1.98	1.10 – 3.58	0.023	1.87	0.49 – 7.10	0.354	1.69	0.93 – 3.05	0.084	0.39	0.01 – 4.38	0.489	6.13	1.76 – 23.95	0.006	5.57	1.57 – 22.41	0.010	2.07	0.78 – 5.50	0.141
Urogenital disorders	2.17	0.76 – 6.51	0.154	0.40	0.00 – 43.29	0.742	1.61	0.58 – 4.41	0.354	-	-	0.999	2.10	0.36 – 9.52	0.365	1.78	0.29 – 8.32	0.489	1.61	0.39 – 5.88	0.484
Other*	5.07	1.99 – 13.76	0.001	12.51	1.09 – 125.70	**0.036**	2.07	0.89 – 4.87	0.091	5.81	0.21 – 168.62	0.304	1.73	0.31 – 7.27	0.481	1.94	0.38 – 8.01	0.384	1.75	0.55 – 5.14	0.322
Vaccination	0.12	0.05 – 0.28	<0.001	0.12	0.03 – 0.37	**<0.001**	0.27	0.12 – 0.57	0.001	0.04	0.00 – 0.22	**0.001**	0.16	0.01 – 0.97	0.098	0.15	0.01 – 0.91	0.085	0.22	0.05 – 0.78	0.028
Previous SARS-CoV-2[5] infection	-	-	-	0.37	0.02 – 3.13	0.425	-	-	-	-	-	0.995	-	-	-	-	-	-	-	-	-

[1] ICU: intensive care units; [2] OR: odds ratio; [3] C.I.: confidence interval; [4] GI: gastrointestinal; [5]SARS-CoV-2: severe acute respiratory syndrome coronavirus 2.

*Other: Skin diseases, genetic disorders, intraocular hypertension, benign intracranial hypertension

Table S4: Multivariate analysis of severe COVID-19[1] (ICU admission, intubation, and death together) in non-Omicron variants among adult participants.

	**Non-Omicron Severe COVID-19 (ICU[2] & Intubation & Death) (N=474)**		
	ORs[3]	C.I.[4]	P-value
Age	1.08	1.04 – 1.12	**<0.001**
Sex (M/F)	3.69	1.34 – 11.21	**0.015**
Autoimmune/ autoinflammatory diseases	7.58	1.07 – 53.63	**0.042**
Dementia/Insomnia/ Neurological disorders	2.03	0.71 – 5.59	0.173
Respiratory system disorders	0.86	0.16 – 3.58	0.842
Malignancies	0.41	0.02 – 3.32	0.472
Hematological disorders	2.45	0.31 – 14.85	0.355
Gastrointestinal system disorders	6.64	1.08 – 36.22	**0.030**
Immunosuppression/Pregnancy	-	-	0.991
Cardiovascular diseases	1.00	0.29 – 3.62	0.998
Metabolic disorders/ endocrinopathies	2.42	0.93 – 6.41	0.070
Urogenital disorders	2.09	0.56 – 7.24	0.255
Other*	2.18	0.71 – 6.32	0.157
Vaccination	0.19	0.04 – 0.66	**0.015**

[1] COVID-19: coronavirus disease 2019; [2] ICU: intensive care unit; [3] ORs: odds ratios; [4] C.I.: confidence interval.

*Other: Skin diseases, genetic disorders, intraocular hypertension, benign intracranial hypertension.

Table S5: Multivariate analysis of hospitalization and pneumonia among unvaccinated adult participants.

N=459 Unvaccinated adults participants	Hospitalization			Pneumonia			ICU[3] admission			Intubation			Death		
	**OR[1]**	**95% C.I.[2]**	**P-value**	**OR**	**95% C.I.**	**P-value**	**OR**	**95% C.I.**	**P-value**	**OR**	**95% C.I.**	**P-value**	**OR**	**95% C.I.**	**P-value**
Age	1.09	1.07 – 1.11	**<0.001**	1.08	1.05 – 1.10	**<0.001**	1.04	1.00 – 1.08	0.063	1.04	1.00 – 1.09	0.059	1.09	1.05 – 1.13	**<0.001**
Sex (M/F)	1.42	0.84 – 2.44	0.195	1.61	0.91 – 2.90	0.104	3.39	0.97 – 14.10	0.067	3.38	0.94 – 14.57	0.075	2.95	1.10 – 8.48	**0.035**
Autoimmune/ autoinflammatory diseases	1.45	0.41 – 4.86	0.550	0.42	0.07 – 1.77	0.276	1.58	0.07 – 12.33	0.708			0.994	1.34	0.14 – 8.87	0.775
Dementia/Insomnia/ Neurological disorders	0.87	0.36 – 2.08	0.762	1.12	0.48 – 2.55	0.791	0.79	0.14 – 3.31	0.764	0.77	0.13 – 3.29	0.742	1.56	0.49 – 4.68	0.435
Respiratory system disorders	2.33	0.86 – 6.51	0.098	4.60	1.77 – 12.35	**0.002**	0.62	0.06 – 3.55	0.629	0.99	0.14 – 4.95	0.987	1.72	0.39 – 6.22	0.436
Malignancies	9.92	1.75 – 81.51	**0.015**	0.61	0.11 – 2.96	0.544	1.79	0.08 – 14.28	0.634	1.50	0.07 – 12.38	0.743	0.36	0.02 – 2.67	0.393
Hematological disorders	3.41	0.61 – 19.18	0.153	1.45	0.21 – 7.78	0.681	1.66	0.07 – 15.18	0.695	3.80	0.35 – 28.04	0.221	3.29	0.40 – 22.68	0.241
Gastrointestinal system disorders	0.35	0.06 – 1.90	0.228	1.21	0.21 – 6.19	0.824	3.01	0.14 – 26.70	0.372	3.07	0.14 – 27.56	0.366	4.84	0.54 – 33.54	0.119
Immunosuppression/Pregnancy	2.74	0.11 – 30.35	0.449	0.00		0.993			0.996			0.998			0.994
Cardiovascular diseases	0.73	0.39 – 1.33	0.310	0.80	0.41 – 1.54	0.512	1.05	0.22 – 5.28	0.952	1.49	0.29 – 9.02	0.644	1.62	0.47 – 6.11	0.454
Metabolic disorders/ endocrinopathies	1.97	1.05 – 3.67	**0.033**	1.90	1.00 – 3.60	**0.049**	4.67	1.33 – 17.55	**0.017**	4.32	1.21 – 16.66	**0.026**	1.56	0.56 – 4.28	0.391
Urogenital disorders	1.34	0.40 – 4.73	0.639	1.04	0.32 – 3.40	0.944	2.34	0.39 – 10.80	0.304	2.12	0.34 – 10.22	0.375	1.22	0.24 – 5.24	0.795
Other*	6.94	2.18 – 25.22	**0.002**	2.58	0.96 – 7.12	0.063	1.78	0.31 – 7.60	0.465	2.01	0.38 – 8.45	0.365	0.81	0.21 – 2.69	0.748
Previous SARS-CoV-2[4] infection	0.62	0.03 – 5.43	0.700	0.00		0.990			0.994			0.996			0.991
Variants (Omicron/Non-Omicron)	0.46	0.18 – 1.10	0.090	0.50	0.18 – 1.23	0.148	0.69	0.03 – 4.65	0.744	1.04	0.05 – 7.41	0.976	1.24	0.26 – 5.02	0.777

[1] OR: odds ratio; [2] C.I.: confidence interval; [3] ICU: intensive care unit; [4] SARS-CoV-2: severe acute respiratory syndrome coronavirus 2.

*Other: Skin diseases, genetic disorders, intraocular hypertension, benign intracranial hypertension

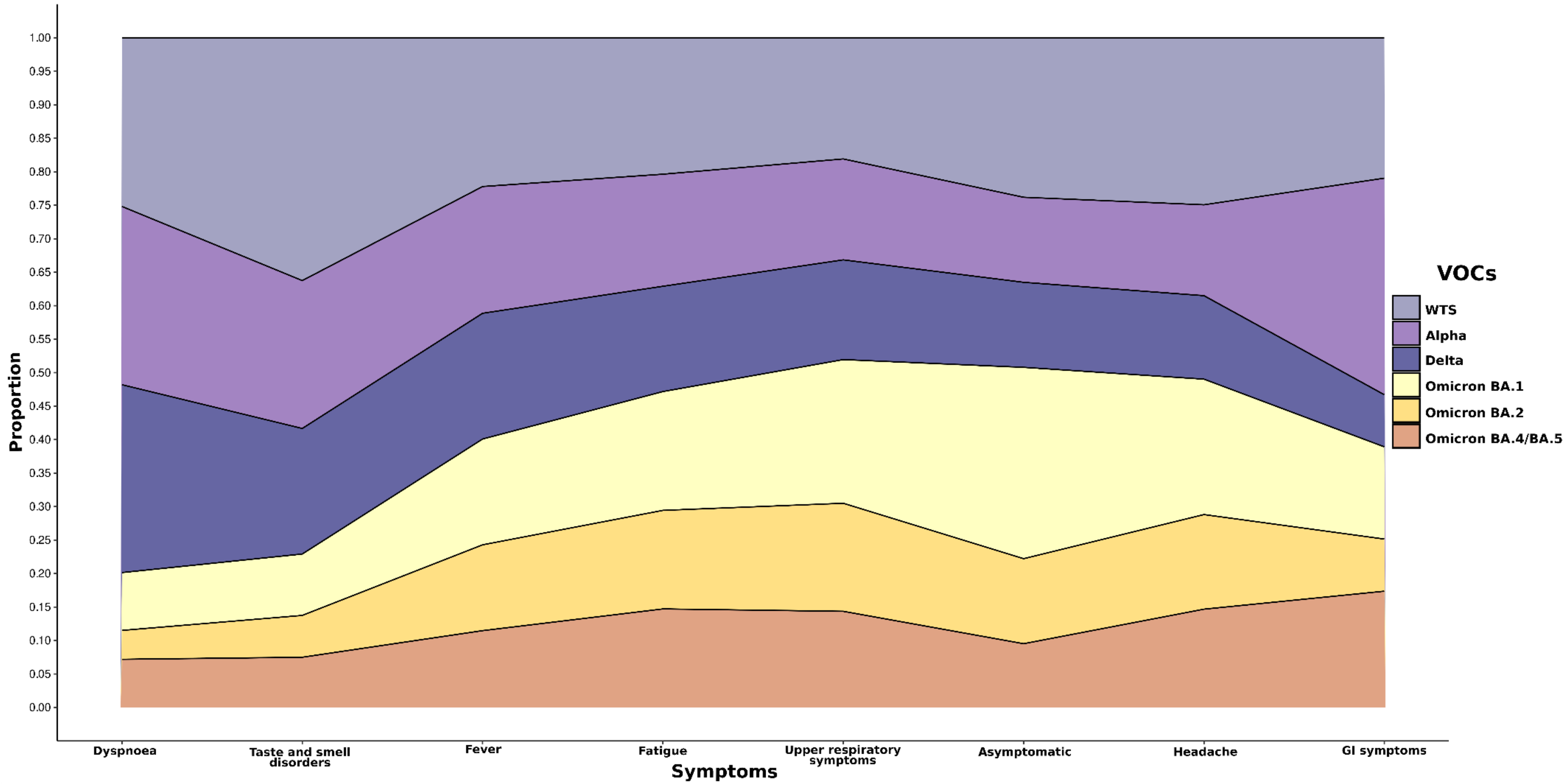

Figure S1: The proportion and the course of symptoms per VOC due to SARS-CoV-2 infection.
